# Modes of Disguising the Odor of Iodoform

**Published:** 1887-08

**Authors:** 


### *MODES OF DISGUISING THE ODOR OF IODOFORM*.*

The odor of iodoform being very offensive to many persons, and at least disagreeable to all, many modes of masking its odor without affecting its therapeutic action have been proposed.

Of this number I have tried the following, which I will number for convenience:

NO. I.

| | |
|---|---|
| Iodoform | 2 parts. |
| Carbolic acid | 1 part. |

This masks the odor of the iodoform completely, but the carbolic acid has the disadvantage of possessing an odor which to many is objectionable.

NO. II.

| | |
|---|---|
| Iodoform | 100 parts. |
| Oil of neroli | 1 part. |
| Oil of lemon | 2 parts. |
| Tinct. benzoin | 2 parts. |
| Acetic acid | 1 part. |

*W. M. Ross.—Read before the Indiana Pharmaceutical Association, at Richmond, June 7 and 8, 1887.

Mix and place in a stoppered flask, and keep at a temperature of from 120° F. to 140° F. over a water bath, for two days.

This process completely covers the odor of iodoform, replacing it by one very agreeable, much resembling cologne. But this process requires considerable time for its completion, which lessens its pharmaceutical value.

NO. III.

| | |
|---|---|
| Iodoform | 15 parts. |
| Powdered charcoal | 10 parts. |
| Camphor | 5 parts. |

This nearly masks the iodoform odor, that of the camphor replac ing it.

NO. IV.

| | |
|---|---|
| Iodoform | 100 parts. |
| Powdered charcoal | 3 parts. |
| Quinine sulph. | 5 parts. |

This is of little or no value. The quinine is useless, and the charcoal is in too small a quantity to be effectual.

NO. V.

| | |
|---|---|
| Iodoform | 1000 parts. |
| Oil of peppermint | 10 parts. |
| Carbolic acid | 5 parts. |

This almost completely masks the iodoform odor, and that of the carbolic acid is rendered much more agreeable by the oil of peppermint.

NO. VI.

Tinct. of tonka beans.

This nearly covers the odor of the iodoform, producing a very agreeable one, slightly resembling that of bitter almonds.

NO. VII.

| | |
|---|---|
| Iodoform | 10 parts. |
| Powdered opium | 1 part. |

This almost completely disguises the odor of iodoform, and in many cases would not be an objectionable addition, but, of course, it cannot be used in prescriptions, unless indicated by the physician.

NO. VIII.

Oil of lavender.

This almost completely masks the iodoform odor by its own, which is very agreeable.

NO. IX.

Oil of sassafras.

Oil of bergamot.

This does very well, much better than the sassafras alone.

NO. X.

Thymol.

This entirely masks the iodoform odor by its own, which, in my estimation, is but little better.

NO. XI.

Iodoform ointment . . . . . . . . . . . 1 troy ounce.

Dilute hydrocyanic acid . . . . . . . 10 drops.

This was tried, and finding it did no perceptible good, I added more of the acid, but with little effect. The odor of the iodoform was still very plain.

NO. XII.

Oil of bitter almonds.

This covers the odor of iodoform by its own almost completely. Some object to the odor of bitter almonds, but, to say the least, it is a decided improvement over that of iodoform.

NO. XIII.

Oil of eucalyptus.

This completely masks the iodoform, but its own odor is far from agreeable.

NO. XIV.

Balsam of Peru.

This almost entirely covers the odor of iodoform, and is always at hand and convenient.

NO. XV.

Roasted coffee.

This masks the odor of iodoform most completely, only its own being perceptible, which is very agreeable. It does not interfere with the medical action of the iodoform in the least, and is every way fitted for this use. I would highly recommend it for this purpose.

NO. XVI.

Chloral hydrate.

By this the odor of iodoform is masked to some extent, but, on being exposed a day or two, returns. The others were not effected perceptibly on being exposed for three days.

I have tabulated these different processes, arranging them on a scale of ten, so as to show, as near as possible, their comparative value, first, as regards the completeness with which they mask the odor of iodoform; second, as to their pharmaceutical value depending on the absence of any therapeutic action, no disagreeable odor, on their convenience, physical properties, etc.:

| PHARMACEUTICAL VALUE. | | COMPLETENESS. | |
|---|---|---|---|
| No. 15 | 10 | No. 15 | 10 |
| No. 5 | 9 | No. 1 | 10 |
| No. 8 | 9 | No. 2 | 10 |
| No. 14 | 9 | No. 10 | 10 |
| No. 8 | 8 | No. 5 | 9 |
| No. 7 | 8 | No. 8 | 9 |
| No. 9 | 8 | No. 9 | 9 |
| No. 12 | 8 | No. 12 | 9 |
| No. 1 | 7 | No. 13 | 9 |
| No. 13 | 7 | No. 6 | 8 |
| No. 2 | 6 | No. 7 | 8 |
| No. 6 | 6 | No. 14 | 7 |
| No. 10 | 6 | No. 16 | 8 |
| No. 16 | 5 | No. 3 | 7 |
| No. 4 | 3 | No. 5 | 3 |
| No. 11 | 1 | No. 11 | 2 |

—*Technics.*

